# Safety profile of the transcription factor EB (TFEB)-based gene therapy through intracranial injection in mice

**DOI:** 10.1515/tnsci-2020-0132

**Published:** 2020-07-15

**Authors:** Zhenyu Li, Guangqian Ding, Yudi Wang, Zelong Zheng, Jianping Lv

**Affiliations:** Department of Neurosurgery, Guangzhou First People’s Hospital, School of Medicine, South China University of Technology, Guangzhou, Guangdong 510180, China

**Keywords:** transcription factor EB, safety, inflammation, adeno-associated virus

## Abstract

Transcription factor EB (TFEB)-based gene therapy is a promising therapeutic strategy in treating neurodegenerative diseases by promoting autophagy/lysosome-mediated degradation and clearance of misfolded proteins that contribute to the pathogenesis of these diseases. However, recent findings have shown that TFEB has proinflammatory properties, raising the safety concerns about its clinical application. To investigate whether TFEB induces significant inflammatory responses in the brain, male C57BL/6 mice were injected with phosphate-buffered saline (PBS), adeno-associated virus serotype 8 (AAV8) vectors overexpressing mouse TFEB (pAAV8-CMV-mTFEB), or AAV8 vectors expressing green fluorescent proteins (GFPs) in the barrel cortex. The brain tissue samples were collected at 2 months after injection. Western blotting and immunofluorescence staining showed that mTFEB protein levels were significantly increased in the brain tissue samples of mice injected with mTFEB-overexpressing vectors compared with those injected with PBS or GFP-overexpressing vectors. pAAV8-CMV-mTFEB injection resulted in significant elevations in the mRNA and protein levels of lysosomal biogenesis indicators in the brain tissue samples. No significant changes were observed in the expressions of GFAP, Iba1, and proinflammation mediators in the pAAV8-CMV-mTFEB-injected brain compared with those in the control groups. Collectively, our results suggest that AAV8 successfully mediates mTFEB overexpression in the mouse brain without inducing apparent local inflammation, supporting the safety of TFEB-based gene therapy in treating neurodegenerative diseases.

## 1 Introduction

Transcription factor EB (TFEB) is a master regulator of the coordinated lysosomal expression and regulation (CLEAR) network in various cell types. The TFEB-activated CLEAR network transcriptionally upregulates the expression of autophagy/lysosome-related genes, leading to the enhanced autophagy/lysosome pathway and subsequent degradation of autophagy/lysosomal substrates, including pathogenic glycosaminoglycans and proteins [1,2]. Lysosomal dysfunction is closely associated with neurodegenerative diseases, such as Alzheimer’s, Parkinson’s, and Huntington’s diseases, and with lysosomal storage disorders caused by the buildup of incompletely degraded metabolites in lysosomes [3,4,5,6]. TFEB-mediated clearance of toxic misfolded proteins has been reported as a promising therapeutic strategy in the treatment of these diseases [6,7,8]; however, there are still safety concerns about clinical application of TFEB due to its potential proinflammatory effects [9,10,11]. Further research is needed to understand the safety of TFEB gene therapy in brain disorders.

Inflammation is a prominent feature of the immune response that is induced by pathogens, irritants, and injured tissues to eliminate these stimuli, leading to compensatory tissue repair and regeneration [12]. Although growing evidence has shown anti-inflammatory roles of the autophagy/lysosome pathway [13,14], several studies also identified its involvement in proinflammatory processes [15,16]. TFEB plays an essential role in autophagy/lysosomal dysfunction during inflammation. In response to macrophage activation by the interaction between pathogens and toll-like receptor (TLR) ligands, the transcript levels of key inflammatory mediators, including interleukin 1β (IL-1β), IL-2, IL-6, IL-27, and tumor necrosis factor (TNF) α, are significantly decreased in TFEB-deficient cells, suggesting that TFEB is required in the innate immune responses in activated macrophages [9,17]. In addition, TFEB induces IL-1β transcription and secretion in collaboration with its binding partner calcineurin in human monocytic cells [18]. Furthermore, a positive correlation between TFEB expression and bladder inflammation has been observed in a mouse model [19]. Although TFEB is a promising therapeutic agent for the treatment of neurodegenerative diseases, it is likely that the proinflammatory features of TFEB might impede its clinical utilization.

To achieve a successful gene therapy, a highly efficient gene delivery system with minimal toxicity and side effects is required [20]. The adeno-associated virus (AAV) is an important gene delivery system because it is nonpathogenic and replication-defective and has the ability to mediate long-term transgene expression *in vivo* [21,22]. To date, there are 11 naturally occurring AAV serotypes and more than 100 engineered AAV variants with different amino acid sequences in the capsid proteins [23,24]. Compared with other serotypes, AAV8 has been reported as a superior serotype for gene delivery to both central and peripheral nervous systems in mice [25,26]. Although AAV vectors have achieved positive outcomes in many diseases [27], the animal studies indicate that AAV components induce the innate immune responses in the host cells [28,29,30,31]. This hinders the clinical application of AAV-mediated gene therapy. Therefore, it is necessary to evaluate the safety of AAV8-mediated TFEB overexpression in the brain.

To assess the safety of AAV8-mediated TFEB overexpression in the brain, we established a mouse model by injecting recombinant AAV8 vectors expressing mouse TFEB gene (mTFEB) in the barrel cortex. The expression of cathepsin B, cathepsin D, and lysosome-associated membrane protein 1 (LAMP1) was determined to evaluate lysosomal biosynthesis induced by TFEB overexpression. The expression of proinflammatory mediators, including TLR4, NF-κB, IL-6, and TNF-α, was examined to assess the inflammatory responses in the brain.

## 2 Materials and methods

### 2.1 Preparation of AAV8 vectors expressing mTFEB cDNA (pAAV8-CMV-mTFEB)

Recombinant plasmids expressing mTFEB (pUC57-mTFEB) were purchased from Biowit, Shenzhen, China. The cloned mTFEB cDNA was digested with *EcoRI* and *HindIII* (New England Biolabs, Ipswich, MA, USA), followed by ligation into pAAV8-CMV vectors (Biowit) using T4 DNA ligase (New England Biolabs). The product was transformed into JM109 competent cells (Biowit). The plasmids were prepared using a miniprep kit (Transgen, Beijing, China) and were transduced into AAV-293 cells using a high-efficiency transfection kit (Biowit) for AAV packaging. The supernatant was collected for preparation of adenoviral particles. An empty vector pAAV8-CMV-ZsGreen (Biowit) was used as a negative control.

### 2.2 Animals

Thirty-six male C57BL/6 mice (6–8 weeks old) were purchased from Guangdong Medical Laboratory Animal Center (Guangdong, China) and were maintained in a specific pathogen-free facility at the Neurobiology Research Center of Zhongshan School of Medicine, Sun Yat-sen University (Guangdong, China), with free access to water and food. The mice were randomly divided into three groups (*n* = 12/group) and stereotaxically injected with 1 μL of phosphate-buffered saline (PBS), pAAV8-CMV-ZsGreen (5 × 10^12^ vg/mL), or pAAV8-CMV-mTFEB (5 × 10^12^ vg/mL) into the barrel cortex. The stereotaxic coordinates from bregma were as follows: anterior–posterior: −1.0 mm, medial–lateral: −2.5 mm, dorsal–ventral: −1.2 mm [32]. The animals were allowed to recover for 2 months before sacrifice. The brain tissue samples (5 mm in diameter and 1 mm in wall thickness) surrounding the injection site were collected from each mouse and stored at −80°C until use.

### 2.3 Ethical approval

The research related to animals use has been complied with all the relevant national regulations and institutional policies for the care and use of animals. All animal experiments were carried out in accordance with the Guide for the Care and Use of Laboratory Animals issued by Sun Yat-sen University.

### 2.4 Immunofluorescence staining

The brain tissue samples were fixed with 4% formalin, embedded in paraffin, and prepared into 30-μm-thick sections. The sections were washed with PBS and immersed in 3% hydrogen peroxide for 5 min, followed by incubation with anti-mTFEB (1:250), anti-GFAP (1:500), and anti-Iba1 (1:500) overnight at 4℃ and with fluorescence-conjugated secondary Ab (1:400) for additional 2 h at room temperature. The images were acquired using an Imager Z1 fluorescence microscopy (Zeiss, Oberkochen, Germany) at magnification 20× and 40×.

### 2.5 Quantitative real-time PCR (qRT-PCR)

Total RNA was isolated from the brain tissue samples using TRIzol reagent (Invitrogen, Carlsbad, CA, USA) and reversely transcribed to synthesize cDNA using a RevertAid first strand cDNA synthesis kit (Thermo scientific, Waltham, MA). PCR was performed using SYBR green (Fermentas, Waltham, MA, USA) and the primers (Sagene, Guangzhou, China) in Table 1.

**Table 1:** Quantitative real-time PCR primers

Gene	Forward primer (5′–3′)	Reverse primer (3′–5′)
TFEB	GCTCAGTGGTCTTGGGCAAA	GTATGGTTGCTCCCATTGTGC
LAMP1	CTTGCACATGGCGCCTCA	ATTCGCAGTCTCGTAGGTGG
CATHB	ACTTAGGAGTGCACGGGAGA	AATCAGGTCATCCGACAGCG
CATHD	TACTCCATGCAGTCATCGCC	GACGACTGTGAAACACTGCG
ACTIN	CACTGTCGAGTCGCGTCCA	ATCCATGGCGAACTGGTGG
TLR4	CACCAGGAAGCTTGAATCCCT	GTCATCAGGGACTTTGCTGAG
NF-κB	CTGTGCCTACCCGAAACTCA	AGGGATGCTGGGAAGGTGTA
IL-6	GAGGATACCACTCCCAACAGACC	AAGTGCATCATCGTTGTTCATACA
TNF-α	CAAAATTCGAGTGACAAGCCTG	ACAAGGTACAACCCATCGGC

TFEB, transcription factor EB; LAMP1, lysosomal-associated membrane protein 1; CATHB, cathepsin B; CATHD, cathepsin D; TLR4, toll-like receptor 4; NF-κB, nuclear factor κB; IL-6, interleukin 6; TNF-α, tumor necrosis factor α.

### 2.6 Western blot assay

The brain tissue samples were lysed with lysis buffer on ice. Tissue lysates were collected by centrifugation at 12,000 rpm for 20 min, followed by heating at 95°C for 5 min in loading buffer. Protein samples were separated by 10% sodium dodecyl sulfate–polyacrylamide gel electrophoresis, transferred to a polyvinylidene difluoride membrane, blocked with 5% skim milk for 1 h, and incubated overnight with primary antibody (Ab) against mTFEB (1:1,000), LAMP1 (Abcam), cathepsin B (Abcam), cathepsin D (Abcam), IL-6 (Cell Signaling Technology), TNF-α (Cell Signaling Technology), NF-κB (ImmunoWay, Plano, TX, USA), or actin (1:5,000) at 4°C. The membranes were washed with tris-buffered saline containing Tween 20 (TBST) three times, incubated with horseradish peroxidase (HRP)-conjugated secondary Abs (R&D Systems, Minneapolis, MN, USA) for 1 h at room temperature, and washed with TBST. The chemiluminescence signal was detected using ImageQuant LAS 4000 mini (GE Healthcare, Chicago, IL, USA) and quantified using Image J (NIH, Bethesda, MD, USA).

### 2.7 Image analysis

For quantification of GFP^+^/RFP^+^ puncta, the images were converted into 8-bit images and binarized after subtracting the background noise using the Image J software, followed by manual verification. The area of RFP^+^ puncta per photo was counted.

### 2.8 Statistical analysis

Data were presented as the mean ± standard deviation. Statistical analyses were carried out using SPSS V21.0 (IBM, Armonk, NY, USA). Statistical significance was analyzed using one-way analysis of variance. A value of *P* < 0.05 was considered statistically significant.

## 3 Results

### 3.1 Successful construction and transduction of AAV8 vectors overexpressing mTFEB

**Figure 1:**
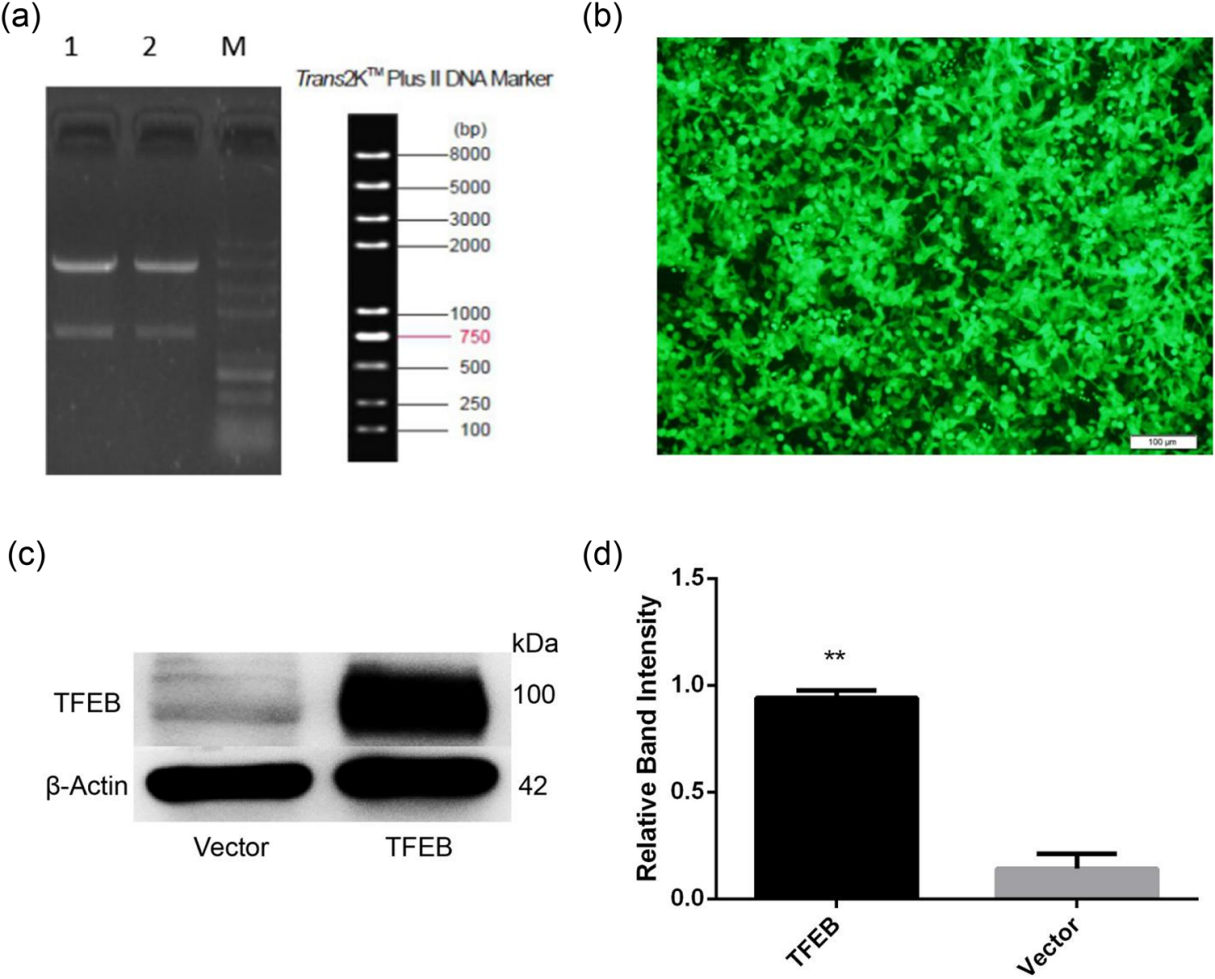
Identification and characterization of mouse transcription factor EB (mTFEB) cDNA. (a) Agarose electrophoresis of digested mTFEB cDNA cloning vector pAAV-CMV-mTFEB (lane 1 and 2). M: DNA marker. A clear band was located at about 1,428 bp, suggesting that the vector was successfully constructed. (b) Fluorescent detection of adeno-associated virus transduction. Scale bar = 100 μm. (c) Western blot analysis of mTFEB expression in infected HEK293 cells with pAAV8-CMV-mTFEB and empty vector. (d) Quantification of (c). **P < 0.01 vs empty vector-injected tissues; *n* = 3. TFEB, transcription factor EB.

To obtain the AAV8 vectors overexpressing mTFEB, we cloned the mTFEB cDNA into the pAAV8-CMV vectors. The agarose electrophoresis showed that a clear band was located between 1,000 and 2,000 bp following enzymatic digestion (Figure 1a), consistent with the size of mTFEB cDNA (1,428 bp) reported in the GenBank database. The sequencing results further confirmed that the cloned fragment was identical to the mTFEB cDNA sequence (data not shown). In addition, green fluorescence was detected in almost all the AAV-293 cells transduced with pAAV8-CMV-ZsGREEN (Figure 1b), suggesting high transduction efficiency of the vectors. Importantly, mTFEB protein expression was dramatically upregulated in pAAV8-CMV-mTFEB-transduced HEK293 cells compared with that in the empty vector-transduced cells (Figure 1c and d), indicating that transduction with mTFEB-overexpressing AAV8 vectors results in TFEB overexpression *in vitro*.

### 3.2 Recombinant pAAV8-CMV-mTFEB vectors induce mTFEB overexpression in the mouse brain

**Figure 2:**
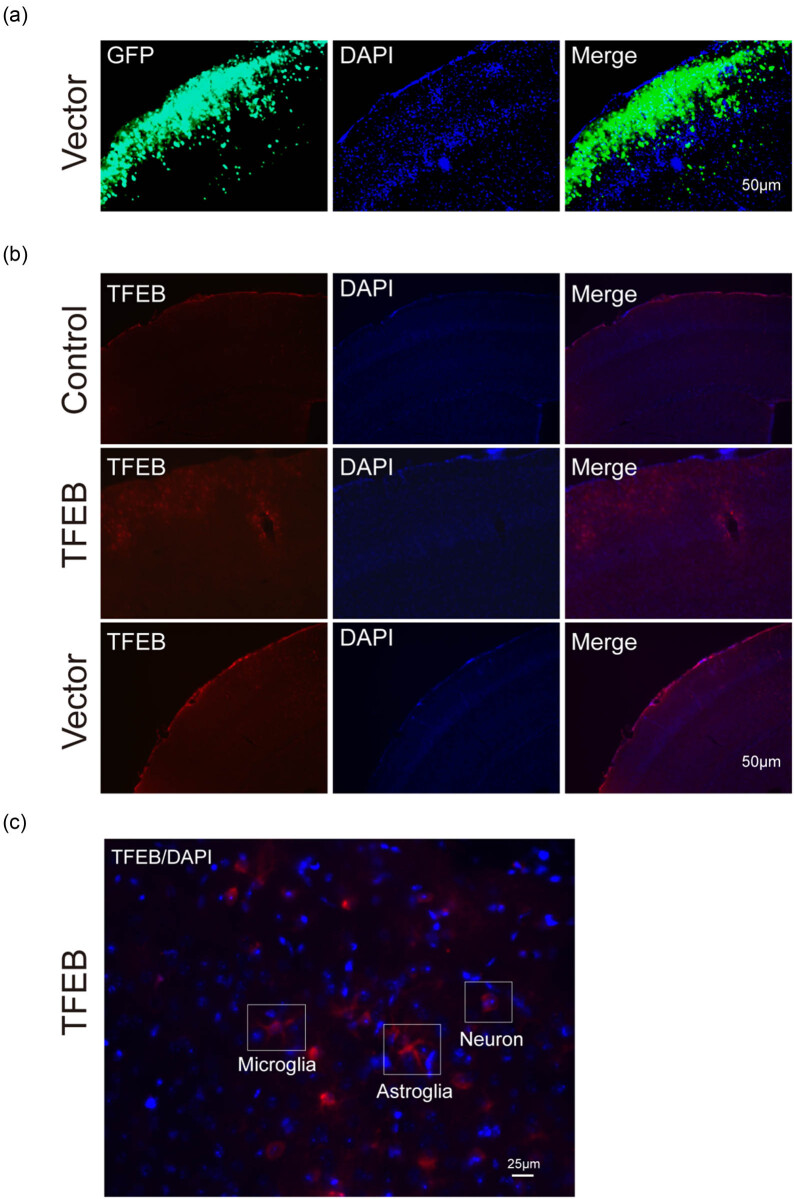
Immunofluorescence staining of mTFEB in mouse brain tissue. Recombinant pAAV8-CMV-mTFEB successfully expresses mTFEB protein in mouse brain tissue. Representative images of brain tissue samples injected with empty vector pAAV8-CMV-ZsGreen (a) and vectors expressing mTFEB, PBS, empty vector (b) are shown. Representative images of mTFEB expressed in different brain cells (microglia, astroglia, and neuron) after mTFEB transfection (c) are shown. Magnification ×10 (a and b) or ×40 (c). Blue represents Hoechst-stained nuclei. Green represents ZsGreen. Red represents mTFEB protein. Scale bar = 20 μm.

To determine whether pAAV8-CMV-mTFEB can express mTFEB protein *in vivo*, we performed immunofluorescence staining in mouse brain tissue samples at 2 months after the injection of vectors. As shown in Figure 2a, compared with that in the empty vector-treated group, mTFEB protein was widely expressed in microglial cells, astrocytes, and neurons in pAAV8-CMV-mTFEB-treated mouse brain (Figure 2b and c), indicating that injection with mTFEB-overexpressing AAV8 vectors effectively induces mTFEB overexpression in the mouse brain.

### 3.3 AAV8-mediated TFEB overexpression upregulates the expression of lysosomal-related genes

**Figure 3:**
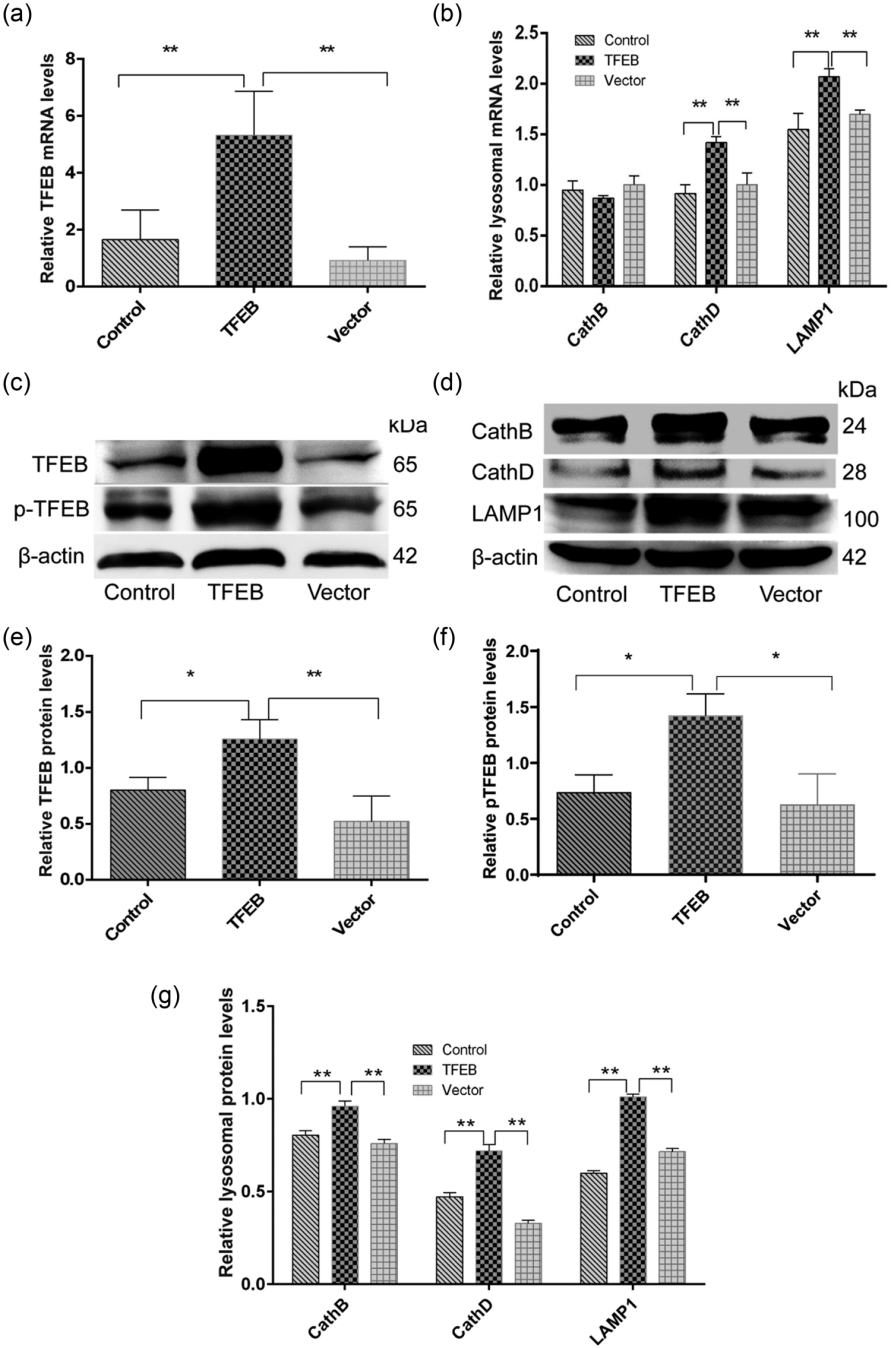
Quantitative real-time PCR (qRT-PCR) and Western blot analyses for TFEB overexpression and lysosomal biogenesis-related genes in brain tissue samples. (a) mRNA expression of mTFEB, (b) cathepsin B, cathepsin D, and LAMP1 was determined by qRT-PCR. (c) Western blot analysis of phospho-mTFEB-Ser211 and mTFEB. β-actin was used as an internal control. (d) Western blot analysis of cathepsin B, cathepsin D, and lysosomal-associated membrane protein 1 (LAMP1). β-actin was used as an internal control. (e) Quantification of mTFEB protein expression. (f) Quantification of p-TFEB-Ser211 protein expression. (g) Quantification of protein expression of cathepsin B, cathepsin D, and LAMP1. *P < 0.05; **P < 0.01 vs PBS- or empty vector-injected tissues; *n* = 3. TFEB, transcription factor EB; CathB, cathepsin B; CathD, cathepsin D; LAMP1, lysosomal-associated membrane protein 1.

To evaluate whether AAV8-mediated mTFEB overexpression promotes lysosomal biosynthesis *in vivo*, we determined the expression of multiple lysosomal biogenesis indicators, including cathepsin B, cathepsin D, and LAMP1 [9]. Real-time PCR (Figure 3a and b) showed that, compared with the control groups, pAAV8-CMV-mTFEB treatment resulted in a significant increase in the mRNA level of mTFEB in mouse brain, along with significant increases in the mRNA levels of cathepsin B, cathepsin D, and LAMP1. Consistent results were observed in the protein expression of mTFEB, phospho-TFEB, cathepsin B, cathepsin D, and LAMP1 (Figure 3c–g). These data suggest that AAV8-mediated mTFEB overexpression promotes lysosomal biosynthesis and activates the macrophage/lysosome pathway in mouse brain.

### 3.4 AAV8-mediated TFEB overexpression does not induce inflammatory response in mouse brain

**Figure 4:**
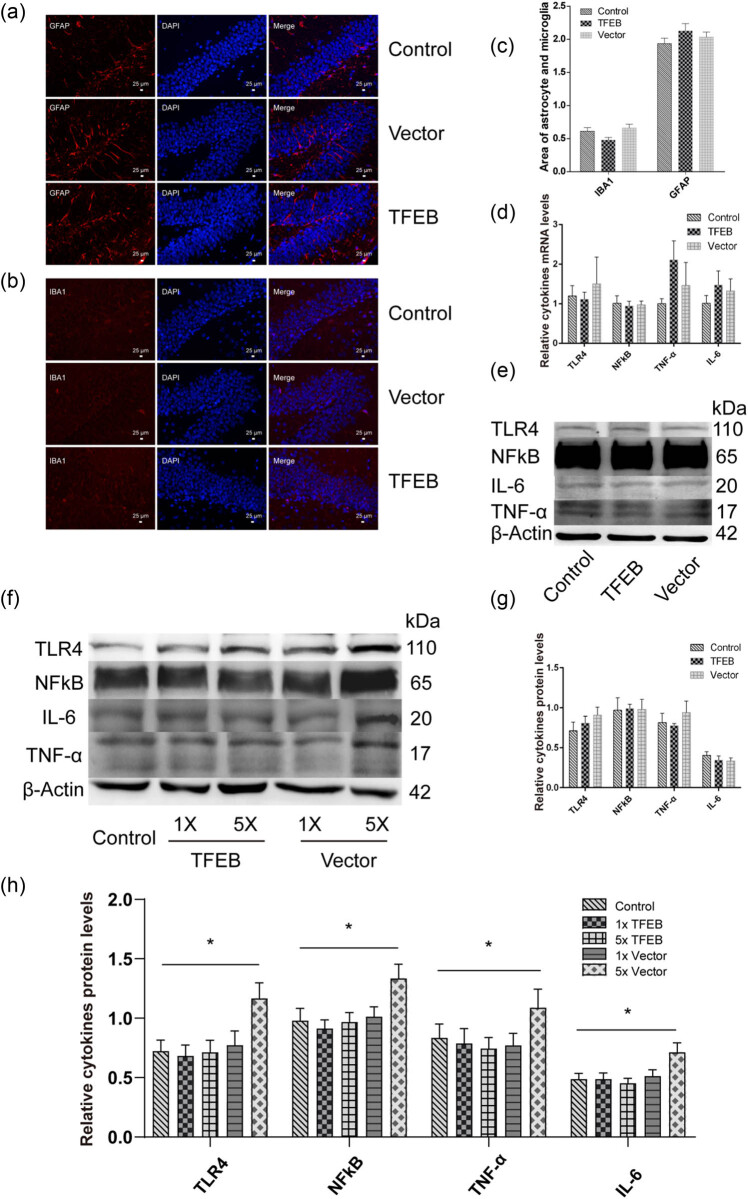
qRT-PCR, Western blot, and immunofluorescence analyses of inflammatory mediators. No significant differences were observed in both GFAP (a) and IBA1 (b) activation between pAAV8-CMV-Mtfeb-treated mouse tissues and control ones. (c) Quantification of the area of GFAP and IBA1 expression. No significant differences were observed in mRNA and protein expression of proinflammatory mediators. (d) mRNA expression of the proinflammatory mediators was determined by qRT-PCR. (e and f) Western blot analysis of toll-like receptor 4, interleukin 6, tumor necrosis factor, and nuclear factor κB. β-actin was used as an internal control. (g) Quantification of (e). (h) Quantification of (f). *n* = 3. TFEB, transcription factor EB; TLR4, toll-like receptor 4; IL-6, interleukin 6; TNF, tumor necrosis factor; NF-κB, nuclear factor κB.

To further investigate whether AAV8-mediated mTFEB overexpression induces inflammatory responses in mouse brain, we examined the expression of proinflammatory mediators. As shown in Figure 4d–h, no significant differences were observed in the mRNA and protein levels of TLR4, NF-κB, IL-6, and TNF-α in mouse brain samples among different groups. Considering the slight upregulation of TNF-α and IL-6 expressions in the mTFEB-overexpressing and empty vectors-treated groups compared with those is the PBS-treated group (Figure 4g), we used higher doses of vectors to further verify the effects of TFEB overexpression on TNF-α and IL-6 expressions. As shown in Figure 4f, no significant changes were observed in the protein levels of TNF-α and IL-6 in the brain samples of mice treated with different doses of AAV8-CMV-mTFEB vectors. However, we observed significant differences in the protein levels of TNF-α and IL-6 in 5× pAAV8-CMV-ZsGREEN-treated mice compared with those in other groups. Next, we evaluated the inflammatory responses in mouse brain using immunofluorescence staining. As shown in Figure 4a–c, no significant differences were observed in GFAP and IBA1 activation among the groups. Taken together, these findings suggest that AAV8-mediated mTFEB overexpression does not induce local inflammation in mouse brain and might serve as a safe gene delivery system in the treatment of neurological disorders.

## 4 Discussion

In this study, we demonstrated that AAV8-mediated mTFEB overexpression did not alter the expression of important proinflammatory mediators in the mouse brain while upregulating the expression of lysosome-related genes. Consistent with our results, Lu et al. have reported that TFEB overexpression inhibits the inflammation in endothelial cells by inhibiting TNFα-induced transcription of IL-1β, IL-8, and IL-6 [33]. In contrast, some studies have shown that TFEB possesses proinflammatory characteristics [9,10,11]. It has been reported that mast cells release tryptase that sequentially activates PAR and TFEB/MiTF signaling in mouse bladder, promoting the synthesis of mMCP-6 and mMCP-7 that induce additional inflammation signals [19].

In our study, we did not observe significant differences in the activation of GFAP and IBA1 or in the expression of TLR4, NF-κB, IL-6, and TNF-α in the brain tissue samples among different groups, suggesting that AAV8-mediated mTFEB overexpression does not induce apparent local inflammation at least in mouse brain. These results are consistent with the study by Lu et al. [33]. Based on these data, we speculate that mTFEB has different functions in different tissues during inflammation. Further study is needed to understand the molecular mechanisms of TFEB during inflammation in different tissues. In summary, AAV8-mediated mTFEB overexpression is a promising and safe approach to activate the CLEAR pathway and thereby targets the misfolded proteins in neurodegenerative diseases without inducing inflammation in the brain.

In the present study, we used the AAV8 serotype to mediate mTFEB overexpression in mouse brain via intraneural injection because AAV8 is superior to other AAV serotypes in gene delivery [25,26]. A previous study has demonstrated that although both AAV1- and AAV8-mediated β-galactosidase overexpression in mouse Schwann cells peaks at 3 weeks after administration, the AAV8-mediated β-galactosidase overexpression is sustained for at least 10 weeks, whereas AAV1-mediated β-galactosidase overexpression markedly declines at 6 weeks after administration [26]. In addition, compared with AAV1 and AAV2, AAV8 is more efficient in gene delivery in various brain regions, including the cerebral cortex, hippocampus, olfactory bulb, and cerebellum. When the same gene with the same copy number is administered, the expression level of AAV8-delivered gene is significantly higher than that of AAV1- or AAV2-delivered gene [25]. Moreover, AAV8 vectors can be intravenously injected into various mouse organs, including the liver, heart, skeletal muscle, and pancreas, with high efficiency [34,35], consistent with our findings showing that AAV8-transduced mTFEB was widely expressed in microglial cells, astrocytes, and neurons. Since TFEB promotes lysosomal biosynthesis, we determined the expression of lysosomal biosynthesis indicators, including cathepsin B, cathepsin D, and LAMP1, to assess the biological function of the exogenous mTFEB [1,2]. Our results showed that the mRNA and protein levels of these indicators were remarkably increased in the mTFEB-overexpressing brain tissue compared with those in the control groups, suggesting that like the endogenous mTFEB, AAV8-delivered exogenous mTFEB also promotes lysosomal synthesis.

Because TFEB and AAV share TLR signaling in inflammation activation [9,28], it is likely that AAV8-mediated TFEB overexpression might exacerbate the inflammatory response. However, in this study, we did not observe significant changes in the expression of important inflammatory cytokines in the mTFEB-overexpressing brain tissue compared with those in the control groups. These findings suggest that AAV8-mediated mTFEB overexpression does not induce inflammation in mouse brain. The possible explanations for the paradox are as follows: (1) despite TFEB- or AAV-induced inflammatory responses have been observed in murine macrophages, human monocytic cells, and mouse bladder and liver [9,19,28], no direct evidence demonstrates that TFEB/rAAV8 induces local immune responses in the brain. The absence of inflammation in the brain in our study is possibly due to tissue heterogeneity. (2) It has been reported that TFEB inhibits antigen presentation via major histocompatibility complex (MHC) class I while enhancing antigen presentation via MHC class II in dendritic cells [10], suggesting that TFEB plays an important role in maintaining the immune system balance. Therefore, the effect of TFEB on inflammatory response is rather complex other than a direct inhibition or promotion. (3) In addition to synthesis and secretion of inflammatory mediators, key features of neural dysfunction also include glial activation, edema, MHC expression, complement activation, expression of adhesion molecules, and invasion of immune cells, which will be investigated in future study.

In conclusion, this study demonstrates that AAV8-mediated mTFEB overexpression in mouse brain upregulates lysosomal biosynthesis-related gene expression without altering proinflammatory cytokine expression. Our results support the feasibility and safety of TFEB-based gene therapy in the treatment of neurodegenerative diseases.
